# The Impact of Conjugation Mode and Site on Tubulysin Antibody‐Drug‐Conjugate Efficacy and Stability

**DOI:** 10.1002/open.202400522

**Published:** 2025-05-28

**Authors:** Sayumi Yamazoe, Qinqin Cheng, Srikanth Kotapati, Vangipuram S. Rangan, Mei‐Chen Sung, Madhura Deshpande, Aarti Jashnani, Cong Qiang, Michael J. Smith, Chin Pan, Gavin Dollinger, Arvind Rajpal, Pavel Strop, Chetana Rao

**Affiliations:** ^1^ Bristol Myers Squibb Research and Development Redwood City California 94063 United States; ^2^ Bristol Myers Squibb Chemical Process Development New Brunswick New Jersey 08901 United States

**Keywords:** antibody‐drug conjugates, deconjugation, payload metabolism, site‐specific conjugations, tubulysin

## Abstract

Antibody‐drug conjugates (ADCs) represent a prominent class of biotherapeutics engineered to selectively deliver cytotoxic payloads to tumors, thereby facilitating targeted cell killing. While first‐generation ADCs, created by conjugating payloads to surface‐accessible lysine or hinge‐cysteine residues, have achieved clinical success, several site‐specific ADCs with defined drug‐to‐antibody ratios are currently under clinical investigation. Herein, the efficacy, stability, and pharmacokinetics of ADCs generated by attaching the drug linker to surface‐exposed lysine residues, hinge‐cysteine residues, and the C’E loop in the CH2 domain (mediated by bacterial transglutaminase) using a tubulysin payload are compared. In N87 xenograft mice, the order of efficacy is C’E loop > hinge‐cysteine > lysine‐conjugated ADCs. Among the three ADCs evaluated, the site‐specific ADC demonstrates superior in vivo stability (minimal payload‐linker deconjugation and limited payload metabolism/deacetylation) and favorable pharmacokinetics (longer half‐life, low clearance, high exposure). In contrast, the lysine‐conjugated ADC exhibits the least stability and poorest pharmacokinetics, which directly correlate with its efficacy. Further investigation into cysteine‐engineered site‐specific ADCs with payloads conjugated at various sites confirms that both the conjugation chemistry and the site of conjugation significantly influence the in vivo stability and pharmacokinetics of site‐specific ADCs.

## Graphical abstract

Two biotransformation events, payload metabolism and deconjugation, are profiled using immuno‐capture ligand‐binding assays and mass spectrometry. The site‐specific antibody‐drug conjugate (ADC) demonstrates superior in vivo efficacy and favorable pharmacokinetics compared to site‐directed or random ADCs.

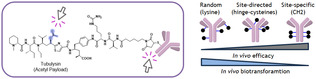

## 1 Introduction

Antibody‐drug conjugates (ADCs) are a proven strategy to treat cancer, with 13 drugs approved by the U.S. Food and Drug Administration (FDA) and over 100 ADCs currently undergoing various stages of clinical research.^[^ ^1^ ^]^ ADCs consist of a cytotoxic payload conjugated to an antibody via a linker. The antibody component of the ADC binds to specific antigens expressed on the tumor cell surface, followed by its internalization and release of the pharmacologically active payload, which ultimately kills the tumor cells.^[^ ^2^ ^]^

The payload‐linker is covalently attached to the antibody by chemical or enzymatic conjugation methods.^[^ ^3^ ^]^ Currently, all FDA‐approved ADCs available on the market are produced via chemical conjugation. In chemically conjugated ADC, the payload‐linkers are attached to surface‐accessible native or engineered amino acid residues on the antibody. The first‐generation ADCs were constructed by attaching the payload‐linker to the native lysine residues, as evidenced in Kadcyla and Mylotarg. The payload‐linker can be attached to 2‐4 hinge‐cysteine amino acids on an antibody by reducing interchain disulfide bonds, as evidenced in Padcev, Tivdak, Adcetris, and Blenrep.^[^ ^4^ ^]^ However, this conjugation results in a heterogeneous mixture of ADCs with drug‐to‐antibody ratio (DAR) ranging from 0 to 8 and narrower therapeutic index than desired.^[^ ^5^ ^]^ Enhertu and Trodelvy belong to the third generation of ADC drugs, which achieved homogeneity by attaching payload‐linkers to all inter‐chain disulfide bonds. These two ADCs have significantly reduced heterogeneity and achieved a higher DAR of ≈8 molecules of cytotoxic agent per antibody compared to early ADCs.

Trends toward generating homogeneous ADCs have gained momentum. In addition to reduction of all interchain disulfide bonds, it is possible to generate site‐specific ADCs with homogeneous DARs by chemically conjugating the payload‐linker to engineered cysteines or non‐natural amino acids, such as *p*‐acetylphenylalanine (pAF), at various positions on the antibody.^[^ ^6^ ^]^ Multiple enzymatic conjugation methods, such as bacterial transglutaminase‐mediated conjugation for generating site‐specific ADCs, have also been developed.^[^ ^7^ ^]^ A number of studies suggest that site‐specific ADCs could achieve superior efficacy compared to lysine‐conjugated or hinge‐cysteine‐conjugated ADCs. This higher efficacy of site‐specific ADCs is attributed to the increased stability of the payload‐linker (reduced deconjugation) in systemic circulation, which ultimately results in improved pharmacokinetics.^[^ ^6^, ^8^ ^]^ However, this improved stability is dependent on the site of conjugation and factors such as solvent accessibility, local environment, and steric hindrance by the antibody domains, and needs to be carefully optimized.^[^ ^9^ ^]^ Furthermore, strategies to minimize payload‐linker deconjugation for first‐generation lysine or hinge‐cysteine ADCs, such as replacing the maleimide linker with an alternative cysteine‐specific electrophile have been shown to offer improved efficacy and stability.^[^ ^10^ ^]^ In another case, it has been shown that heterogeneous conjugates have similar in vitro and in vivo properties to homogeneous conjugates, highlighting the importance of careful evaluation of the antibody, payload, linker, conjugation chemistry, and conjugation site to determine the optimal ADC.^[^ ^11^ ^]^

Though substantial research has been conducted to address the consequences of conjugation site‐specificity on ADC attributes,^[^ ^5^, ^12^ ^]^ to the best of our knowledge, a study comparing the efficacy, stability, payload metabolism, and pharmacokinetics of ADCs produced by the three major conjugation modes (surface lysine, hinge‐cysteine, and enzyme‐mediated site‐specific approaches) is still limited. To answer the above questions, we compared the preclinical efficacy and biotransformation of ADCs (with the same cathepsin B cleavable linker and tubulysin payload) prepared by different conjugation methods, either chemical or enzyme‐based. These methods included lysine (2‐iminothiolane mediated), hinge cysteine (interchain disulfides), and a bacterial transglutaminase‐mediated site‐specific method. The in vivo biotransformation study was enabled by the discovery of anti‐drug antibodies designed to distinguish intact and deacetylated tubulysin payload. We observed that, at the same dose levels, the site‐specific ADC with drugs attached to the CH2 domain demonstrated improved in vivo efficacy compared to the hinge‐cysteine‐modified ADC or the lysine‐conjugated ADC. Additionally, site‐specific ADCs exhibited better in vivo stability, with reduced deconjugation and payload metabolism/deacetylation, and showed superior pharmacokinetics compared to lysine or hinge‐cysteine conjugated ADCs. This improved stability directly correlated with their enhanced efficacy. Extending our investigation from stochastic interchain disulfide‐modified ADCs to engineered cysteine‐based site‐specific ADCs, we found that the site of conjugation affects tubulysin payload deacetylation as well as payload‐linker deconjugation.

## 2 Results and Discussion

### 2.1 ADC Preparation

**Figure 1:**
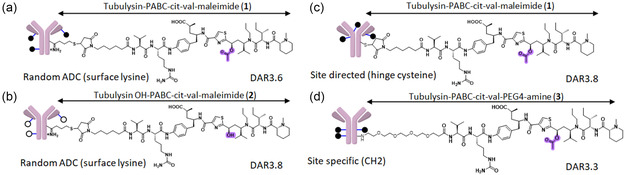
Different conjugation strategies: a,b) 2‐IT and subsequent maleimide chemistry‐based conjugation to introduce tubulysin payload into surface‐exposed lysine residues. Tubulysin payload‐linker (**1**) or deacetylated tubulysin payload‐linker (**2**) was attached to aMeso to produce aMeso (lysine) ADC with intact tubulysin (a) and aMeso (lysine) ADC with deacetylated tubulysin (b), respectively. c) aMeso (hinge‐cysteine) ADC was made by reduction of hinge disulfides followed by payload conjugation to free thiols. d) bTGase‐mediated conjugation of tubulysin payload‐linker (**3**) to Q295 and Q297 residues on N297Q mutated mAb yielded aMeso (N297Q) ADC.

We used ADCs targeting mesothelin (Meso), a glycosylphosphatidylinositol‐anchored cell surface protein, as mesothelin represents a high‐value tumor‐associated target expressed in ≈30% of all cancers.^[^ ^13^ ^]^ ADCs were prepared using different modes of conjugation and conjugation chemistries. The detailed synthesis, conjugation, purification, and characterization methods are described in the supporting information section. Lysine‐conjugated random ADCs: tubulysin‐aMeso ADC and deacetyl tubulysin‐aMeso ADC were prepared by thiolation of a human antimesothelin monoclonal antibody with 2‐iminothiolane (2‐IT), followed by conjugation with tubulysin‐PABC‐cit‐val‐maleimide (**1**) and its deacetylated analogue (**2**), respectively (lysine ADC) (**Figure** **1**a,b). Hinge‐cysteine‐conjugated ADC (hinge‐cysteine ADC) was generated by reduction of interchain disulfide bonds, followed by conjugation with **1** (Figure 1c). A tubulysin payload linker with an amine handle and a PEG spacer (**3**) was conjugated to an anti‐Meso antibody with an N297Q mutation using bacterial transglutaminase (bTG), forming a site‐specific ADC with payloads at the 295 and 297 positions, resulting in a theoretical maximum drug‐to‐antibody ratio (DAR) of 4 (N297Q ADC) (Figure 1d). Lysine‐ and hinge‐cysteine‐based ADCs were also generated with a target DAR of 4 for comparison with the N297Q ADC. Multiple site‐specific ADCs were also prepared by chemical conjugation of **1** to specific cysteines engineered at various positions (T169C, S239C, T289C, P343C, Q362C, and K414C) on the anti‐Meso antibody for subsequent in vitro and in vivo studies. The generated ADCs were tested for their binding characteristics against three cell lines expressing different levels of the Meso target, along with a cell line lacking expression of the antigen. The ADCs demonstrated target‐dependent cellular binding comparable to the naked antibody, suggesting that drug attachment did not alter binding characteristics (Supporting Figure S1, Supporting Information). To evaluate the cell‐killing activity of the ADCs prior to in vivo functional characterization, the ADCs were subjected to cytotoxicity assays. The ADCs with higher drug load showed greater activity against the H226 cell line, which is less sensitive to the payload, and the N87 cell line, which has low antigen expression. In contrast, all ADCs showed similar activity against OVCAR3, which is sensitive to tubulysin and expresses a high level of the antigen (Supporting Figure S2, Supporting Information).

### 2.2 Effect of Deacetylation of Tubulysin Payload on ADC Efficacy

Previous studies have shown that the conjugated tubulysin payload undergoes deacetylation (hydrolysis of acetate ester) in vitro and in vivo.^[^ ^12^ ^]^ Furthermore, it has been reported that an ADC conjugated with a deacetylated payload has shown a complete loss of activity in an in vitro cell cytotoxicity assay.^[^ ^12^ ^]^ To understand the effect of this biotransformation on ADC activity, we prepared a pair of ADCs containing acetylated and deacetylated ADCs targeting Meso, using 2‐IT‐mediated bioconjugation. Both ADCs displayed similar DAR based on liquid chromatography‐mass spectrometry (LC‐MS) analysis: 3.6 and 3.8 for intact tubulysin and deacetylated tubulysin ADCs, respectively. Consistent with what is reported, the ADC armed with acetylated tubulysin (**1**) demonstrated dose‐dependent activity, while the ADC made with deacetylated tubulysin (**2**) failed to show any activity (Supporting Figure S3, Supporting Information). Activity was also dependent on the targeting arm, as evidenced by the lack of activity seen with the nontargeted control antibody‐based ADC. We aimed to further understand the consequence of the deacetylation of the tubulysin payload class‐based ADC in an in vivo efficacy study. We therefore compared the efficacy of anti‐Meso lysine‐modified ADCs bearing intact tubulysin and deacetylated tubulysin in an N87 gastric xenograft model expressing the Meso target endogenously (Supporting Figure S4, Supporting Information). When the animals were dosed at 3.5 mg/kg, single dose, we observed tumor regression only with the anti‐Meso ADC bearing **1** and not with the deacetyl ADC produced with **2** (Supporting Figure S4, Supporting Information). This clearly demonstrated and confirmed the requirement of the acetate group for the activity of this payload.

**Figure 2:**
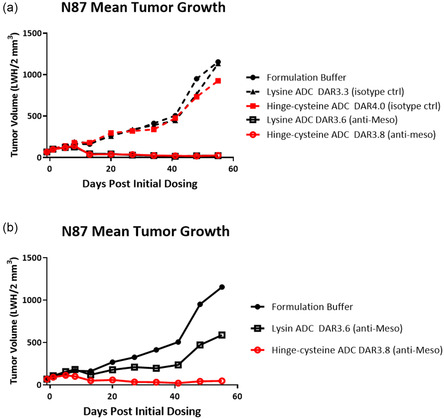
In vivo efficacy of anti‐Meso ADCs made with two different modes of bioconjugation. When tumors reached 100 mm^3^, mice were treated with a single injection of either 3.5 mg/kg of anti‐Meso or isotype control ADCs. a) ADCs demonstrated a targeting arm‐dependent activity to inhibit tumor growth. b) At low dose level of 1 mg/kg, two ADCs showed a distinct in vivo efficacy.

**Figure 3:**
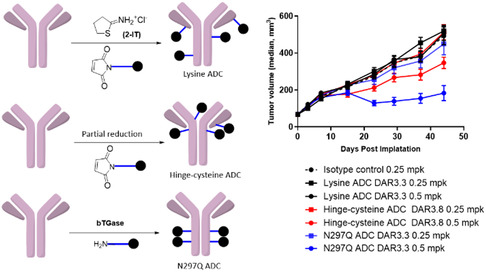
Comparison of in vivo efficacy of the lysine, hinge‐cysteine, and N297Q ADCs targeting Meso‐dosed i.v. at 0.25 and 0.5 mpk in N87 xenograft model mice.

Dose range finding studies were conducted with anti‐Meso ADCs made by lysine (2‐IT) and hinge‐cysteine (interchain disulfide) conjugation methods. The efficacy of these two ADCs was evaluated in an N87 gastric in vivo xenograft model at 1 and 3.5 mg/kg i.v. dose. Both ADCs targeting Meso exhibited substantial in vivo efficacy, which was not observed with the administration of isotype control ADCs, demonstrating target‐dependent tumor growth inhibition (**Figure** **2**a). At the 3.5 mg/kg dose level, we saw efficacy in both modalities with little differentiation. However, we observed a clear distinction in efficacy between the two modes of conjugation at a lower dose level (Figure 2b). At a dose of 1 mg/kg, the site‐directed ADC with drugs attached to hinge cysteines demonstrated higher efficacy compared to the lysine‐modified ADC (Figure 2b). Based on this observation, we performed a study to compare the in vivo attributes of three differently conjugated ADCs—lysine, hinge‐cysteine, and N297Q ADCs—using the same in vivo xenograft model at 0.25 mg/kg and 0.5 mg/kg. The three ADCs showcased well‐differentiated activity in this model (**Figure** **3**). The most prominent efficacy was observed with the site‐specific ADC (N297Q) dosed at 0.5 mg/kg, followed by the hinge‐cysteine ADC dosed at 0.5 mg/kg (Figure 3). The lysine‐conjugated ADC did not demonstrate efficacy at any of the dose levels evaluated in the study. We observed moderate efficacy of the N297Q ADC at the lower dose of 0.25 mg/kg (Figure 3). The administration of the ADCs was well tolerated, and none of the three ADCs had a significant impact on the body weight of the treated animals (Supporting Figure S5, Supporting Information).

### 2.3 Pharmacokinetic (PK) Analysis

To further investigate the observed differences in efficacy between the three ADCs, serum samples from the above efficacy study were analyzed to determine the pharmacokinetics. One limitation of using mouse models to test ADC pharmacokinetics is the overexpression of carboxylesterase 1c (ces1c) in mice.^[^ ^14^ ^]^ Ces1c is an enzyme that can significantly influence the metabolism and stability of ADCs. The presence of ces1c in mice can lead to differences in the breakdown and clearance of ADCs compared to humans, who do not express this enzyme in the same way. As a result, the pharmacokinetic data obtained from mouse models may not accurately predict the behavior of ADCs in humans. The PK of the random ADC we generated with the tubulysin linker payload was extensively studied in nonclinical species, including mouse, rat, and monkey, as well as human patients (Table S1, Supporting Information).^[^ ^15^ ^]^ The PK profile of the ADC was found to be consistent between different species; therefore, we believe that the data generated in the mouse models provide substantial translational value.

**Figure 4:**
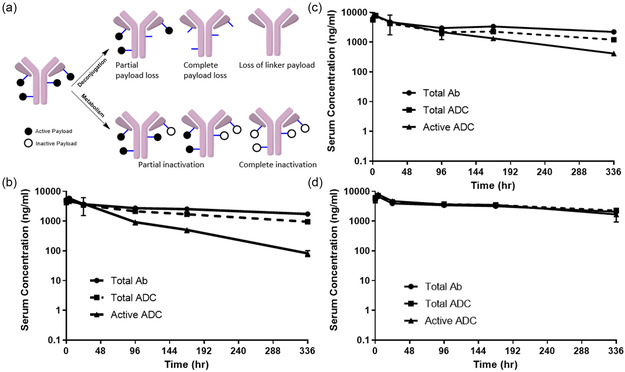
a) Representative scheme of possible ADC metabolites in vivo, b) concentrations of total Ab (antibody with or without conjugated payload), total ADC (antibody with conjugated active and inactive payload), active ADC (antibody with conjugated active payload) in mice dosed with 0.5 mg/kg of aMeso (lysine) ADC, c) aMeso (hinge cysteine) ADC, and d) aMeso (N297Q) ADC.

To enable a versatile bioanalytical approach to track ADC stability, we developed specific ligand‐binding assays (LBA) reagents to distinguish intact and deacetylated payload‐bearing ADC species in the serum samples. Tubulysin ADC can undergo several biotransformations in vivo: deconjugation of the payload and payload‐linker, and payload deacetylation (inactive payload) (**Figure** **4**a). We produced specific reagents that could differentiate between deconjugation and payload deacetylation events. Through mouse immunization with a payload‐attached immunogen, we identified two anti‐idiotypic mouse reagent mAbs for LBA, each of which recognizes a unique species of the payload (Supporting Figure S6, Supporting Information). For example, LBA‐R1 recognizes both intact tubulysin payload and deacetylated tubulysin (total ADC species). LBA‐R2 is specific for intact tubulysin (active ADC species) and not deacetylated tubulysin (inactive ADC species). Both LBA‐R1 and R2 demonstrate binding to N‐alkylation analogues of tubulysin (ADC‐3); however, these two reagents display distinct recognition patterns for the acetyl group at the C11 position, indicating that R1 and R2 have overlapping yet different epitopes (Supporting Figure S6, Supporting Information). In contrast, neither R1 nor R2 showed binding to the parental antibody, suggesting that these binders are specific to a certain chemical motif present in the tubulysin scaffold (Supporting Figure S6, Supporting Information). The discovery of the LBA‐R1 and LBA‐R2 reagents enabled us to quantify the total ADC and active ADC species, respectively, in serum samples. Furthermore, total Ab (with or without the payload) was also quantified and compared against the total ADC to determine the degree of deconjugation.

The total Ab, total ADC, and active ADC levels in mice dosed with the three different ADCs (dosed at 0.5 mg/kg) were determined using LBA, and the PK profiles were generated (Figure 4b–d, **Table** **1**). For the lysine‐ and hinge‐cysteine‐modified ADCs, the discrepancy between the levels of total Ab and total ADC, as well as the difference between total ADC and active ADC species, increased from 0 to 336 h, indicating increased deconjugation and payload deacetylation over time (Figure 4b,c). However, the N297Q ADC displayed a negligible discrepancy among the three analyte curves, indicating minimal deconjugation and payload deacetylation compared to the other two ADCs (Figure 4d). AUC, CL/F, and t1/2 of total antibody species are similar for all three ADCs (Table 1). The exposure of active ADC species is highest for the site‐specific (N297Q) ADC, followed by the hinge‐cysteine modified ADC, showing a fourfold and twofold higher shift compared to the lysine‐conjugated ADC, respectively. The clearance of active ADC species was highest for the lysine‐conjugated ADC: fivefold compared to the N297Q ADC and twofold compared to the hinge‐cysteine ADC. The t1/2 of active ADC species for N297Q (bTG) ADC (326 h) is higher compared to the hinge‐cysteine ADC (100 h) and lysine‐conjugated ADC (68 h).

**Table 1:** PK parameters of three ADCs dosed at 0.5 mg/kg in vivo.

	Lysine ADC			Hinge‐cysteine ADC			N297Q ADC		
	Total Ab	Total ADC	Active ADC	Total Ab	Total ADC	Active ADC	Total Ab	Total ADC	Active ADC
Cmax (ng/ml)	5,868	5,436	4,639	7,904	7,859	7,610	6,702	7,229	8,009
AUC (0–336 h) (μg*h/ml)	891	663	340	1,115	809	651	1,065	1,172	1,133
T½ (h)	351	205	68	330	192	100	337	334	326
CL/F (ml/h/kg)	0.28	0.53	1.43	0.23	0.44	0.70	0.24	0.22	0.30
% Deconjugation	25%			27%			<0.1%		
% Payload deacetylation	48%			24%			3%		

By comparing the levels of total antibody, total ADC, and active ADC, we can infer the levels of deconjugation and payload metabolism for the ADCs. The observed level of deconjugation (comparing AUC of total Ab and total ADC) was 25% for the lysine ADC, 27% for the hinge‐cysteine ADC, and less than 0.1% for the N297Q ADC. The observed level of payload metabolism (comparing AUC of total ADC and active ADC) was 48% for the lysine ADC, 24% for the hinge‐cysteine ADC, and 3% for the N297Q ADC. In summary, the site‐specific N297Q ADC had the best in vivo stability, followed by the hinge‐cysteine ADC and the lysine‐conjugated (2‐IT) ADC, which correlates with the in vivo efficacy of these ADCs. The site‐specific N297Q ADC generated via bTGase conjugation had negligible deconjugation and deacetylation compared to the lysine and hinge‐cysteine ADCs (Table 1). This can be attributed to the nature of the chemical bonds and the conjugation site. The bTGase ADC is generated by the formation of an isopeptide bond between the glutamine side chain and the payload linker with an amine handle. In contrast, the lysine (2‐IT) and hinge‐cysteine ADCs contain thiosuccinimide linkages that can undergo deconjugation, resulting in the loss of the payload‐linker via the formation of covalent adducts with serum components such as glutathione, cysteine, and albumin.^[^ ^16^ ^]^ The conjugation sites at the Q295 and Q297 positions of the N297Q ADC also provide a shielding effect compared to more solvent‐exposed residues. Our previous study also suggested the potential interaction of the linker‐payload with the inner portion of the CH2 domain, as seen with aglycosylated antibodies such as N297Q ADC, which may impact ADC stability.^[^ ^17^ ^]^ While it is challenging to completely separate the contributions of conjugation chemistry and site to the in vivo activity of ADCs, our findings suggest that the mode of conjugation does influence the in vivo stability of ADCs. However, it is important to acknowledge the limitations of this study, as the interplay between conjugation chemistry, site‐specific effects, and the presence or absence of carbohydrate chains is complex and may not be fully captured in our current analysis in a controlled manner. To validate the analytical method enabled with LBA reagents specific to each chemical motif, we also performed biotransformation analysis using mass spectrometry combined with immunocapture enrichment (Supporting Figure S7, Supporting Information).^[^ ^18^ ^]^ To increase mass accuracy and particularly enable tracking of the biotransformation events, IdeS digestion‐mediated sub‐fragment analysis was performed. For the lysine‐conjugated ADC, the data of both the Fc and Fab portions were shown. Both sub‐fragments showed significant deconjugation and deacetylation, as evidenced by reduced levels of peaks corresponding to drug‐linker attached species, as well as mass conversion of the observed peak over time (Supporting Figures S7a,b, Supporting Information). The mass profile of hinge‐cysteine‐labeled ADC showed a lesser degree of deconjugation, suggesting enhanced stability over the lysine‐conjugated ADC (Supporting Figure S7c, Supporting Information). Finally, the site‐specific ADC with N297Q mutation demonstrated the highest stability: the ADC remained mostly intact up to 96 h (Supporting Figure S7d, Supporting Information). These data generated with an alternative quantitation method align fairly well with the data obtained with LBA reagents.

### 2.4 Comparison of In Vitro and In Vivo Stability of Cysteine‐Engineered Site‐Specific ADCs

**Figure 5:**
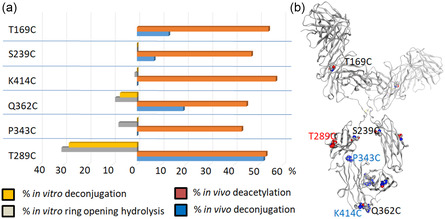
Stability of cysteine‐engineered site‐specific ADCs. a) % deconjugation and % succinimide hydrolysis (left) observed for ADCs of interest after incubation in 500 uM glutathione for 5 days or pH9 buffer for 2 days. % deacetylation and % deconjugation observed in vivo (right). b) Position of engineered cysteines.

We then proceeded to investigate the effect of the site of conjugation on the in vivo stability of tubulysin ADCs. To evaluate this, we generated six site‐specific ADCs with tubulysin‐PABC‐cit‐val‐maleimide (**1**) (the same payload‐linker as in the hinge‐cysteine ADCs) conjugated to cysteines engineered at different positions on the anti‐mesothelin antibody using cysteine‐maleimide chemistry. These ADCs were then incubated in mouse serum and analyzed by affinity capture LC‐MS to evaluate their in vitro serum stability. The percentage of in vitro payload deacetylation (96 h) ranged between 8% and 14% for all ADCs except T169C ADC, which had no in vitro deacetylation (**Figure** **5**). The percentage of in vitro deconjugation (96 h) varied depending on the site of conjugation: S239C ≈ K414C (0%) < P343C < T169C < Q362C < T289C (40%) (Figure 5, Supporting Figure S7e–j, Supporting Information). These ADCs were subsequently dosed intravenously at 2 mg/kg in nontumor‐bearing SCID mice, and the serum samples were analyzed by LBA, and the PK parameters were generated (Supporting Figure S8, Supporting Information). The percentage of payload metabolism for all six ADCs varied between 45% and 60%. The percentage of deconjugation differed significantly depending on the site of conjugation: P343C, K414C (< 0.1%) < S239C < T169C < Q362C < T289C (52%) (Figure 5).

As shown in Figure 5, there is a good correlation between the in vitro and in vivo stability of these ADCs with respect to payload‐linker deconjugation. T289C and Q362C ADCs were the least stable (highest percentage of deconjugation), and K414C ADC was the most stable in vitro and in vivo. Several studies have reported that succinimide hydrolysis and ring opening prevent deconjugation of the payload‐linker and improve ADC stability.^[^ ^12^, ^16^ ^]^ We observed that the percentage of in vitro succinimide hydrolysis was S239C ≈ K414C (0%) < T169C < P343C < Q362C < T289C (63%). Surprisingly, we observed that K414C ADC is the most stable ADC despite negligible succinimide hydrolysis, while T289C and Q362C ADCs were the least stable (highest percentage of deconjugation), which exhibited the highest percentage of succinimide hydrolysis.

Overall, we observed a disconnect between succinimide hydrolysis and ADC stability, which aligns with previous studies suggesting that succinimide hydrolysis is not the sole factor contributing to high serum stability. Instead, the instability of ADCs appears to be influenced by a variety of factors, including solvent accessibility, attachment site, cysteine thiol pKa, local charge environment, hydrophobicity, and potential binding to serum albumin.^[^ ^12^, ^18^, ^19^ ^]^ There was no clear difference in deacetylation levels for these site‐specific ADCs bearing payloads in different positions on the antibody.

### 2.5 Comparison of In Vitro and In Vivo Attributes of Random and Site‐Specific ADCs with PBD Payload Class

**Figure 6:**
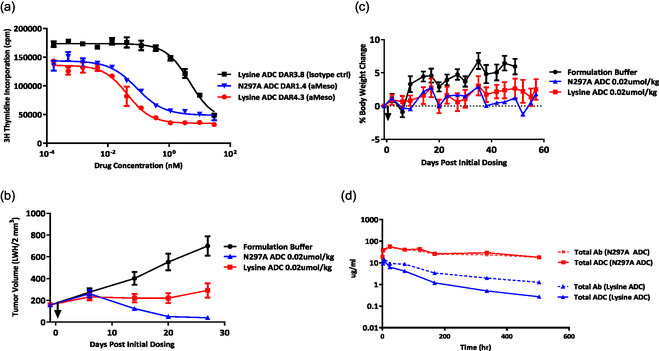
In vitro and in vivo characterization of PBD‐ADCs constructed with random or site‐specific conjugation chemistry. a) In vitro cell killing activity of PBD ADCs against the N87 cell line expressing the Meso target. Concentration was normalized based on payload molarity. The ADCs showed targeting arm‐dependent activity. b) In vivo efficacy of ADCs dosed via IP at drug equimolar levels (1 mg/kg for lysine, 3 mg/kg for N297A ADC). Site‐specific N297A ADC showed greater activity. c) Body weight loss data after drug administration. No substantial difference between the two ADCs was observed. d) Concentrations of total Ab and total ADC in mice dosed with 0.02 μmol/kg of aMeso (lysine) ADC and aMeso (N297Q) ADC.

To assess if the observed findings with the tubulysin payload are general, we extended the comparison study to another class of payload. Pyrrolobenzodiazepine (PBD) payloads represent a major class of cytotoxic agents used in ADCs due to their potent antitumor activity. PBDs are derived from naturally occurring antitumor antibiotics and are known for their potent DNA‐interacting properties. We generated the ADCs leveraging random, site‐directed, and site‐specific conjugations with PBD dimer‐based linker payloads with maleimide or amine handles (Supporting Figure S9, Supporting Information). Analogous to tubulysin‐ADC generation, the PBD linker‐payload was conjugated to the aMeso antibody using chemical or transglutaminase‐mediated conjugations. Due to the high hydrophobicity of this payload class, attaching the linker payload to the interchain disulfide region was not successful. The conjugated products showed a high propensity to form high‐order species. This is presumably due to the enrichment of hydrophobic payloads in close proximity, which can create large hydrophobic patches. The random ADC and site‐specific ADCs were produced successfully, and thus these two ADCs were subjected to further functional characterization. Both ADCs showed similar levels of cell‐killing potency against the N87 cell line expressing the Meso target (**Figure** **6**a). However, a significant difference manifested in in vivo studies; the PBD ADC loading payload at the CH2 domain (N297A ADC) demonstrated substantially enhanced in vivo efficacy at a drug equimolar dose level (0.02 μmol/kg) (Figure 6b). Both ADCs showed similar effects on the body weight of treated mice (Figure 6c). As expected, the N297A ADC demonstrated higher in vivo stability (Figure 6d). The negligible separation of total ADC and total mAb was observed with the site‐specific ADC, indicative of high resistance against deconjugation. In contrast, the random ADC showed a discrepancy between total ADC and total mAb, suggesting deconjugation of the ADC over time. The data generated with the PBD payload class was consistent with the findings with the tubulysin payload.

## 3 Conclusion

In the current study, we investigated the relationship between conjugation mode (lysine, hinge‐cysteine, and N297Q), efficacy, in vivo stability (deconjugation and payload metabolism), and pharmacokinetics of tubulysin ADCs. We observed that the site‐specific N297Q ADC generated by bTGase chemistry had the best efficacy, followed by the hinge‐cysteine ADC. The lysine‐conjugated ADC was the least potent and showed no difference compared to the isotype control at the dose levels evaluated. The site‐specific ADC, with drug linkers attached to the Q295 and Q297 positions, had the best in vivo stability (negligible payload‐linker deconjugation and minimal payload deacetylation) and pharmacokinetics, followed by the hinge‐cysteine ADCs (moderate deconjugation and payload deacetylation). Lysine‐conjugated ADCs had poor in vivo stability and pharmacokinetics (moderate deconjugation and high payload deacetylation). The detailed profiling of in vivo biotransformation of the ADCs, including deacetylation of the payload, was enabled by leveraging newly generated antidrug antibodies specific for active ADC species. The results demonstrate the feasibility of generating antibodies that can distinguish the presence of one acetyl group. The bioanalytical data obtained with newly discovered anti‐drug reagents that can distinguish the acetyl group on the payload were further validated with immuno‐capture mass spectrometric analysis. Overall, we observed an excellent correlation between the stability, pharmacokinetics, and efficacy of ADCs. These reagents could be valuable for tracking the metabolic conversion of the payload component of the ADC using an LBA assay, which could facilitate the bioanalytical characterization of this complex modality. We further observed that the levels of payload‐linker deconjugation and payload deacetylation varied depending on the mode and site of conjugation. The characterization was extended to another payload class, pyrrolobenzodiazepine (PBD), which is commonly used in ADC design. The data were in alignment with what was observed with ADCs constructed using the tubulysin payload class. Site‐specific ADCs demonstrated greater in vivo efficacy compared to randomly conjugated ADCs. Though mice are known to overexpress carboxylesterase, which induces species discrepancies in ADC stability, we believe the observed deconjugation kinetics and metabolic conversion rate provide significant translational value, based on the consistency of PK parameters generated with the lysine ADC across multiple preclinical species and human patients. All studied ADCs were well tolerated in mouse models, as evidenced by no significant impact on mouse body weight, regardless of differences in ADC design. Further study is warranted to address their overall impact on therapeutic indices.

There was no correlation between ADC thiosuccinimide hydrolysis and stability. These findings emphasize the importance of considering the totality of ADC design, as the study results suggest that in vivo behaviors, including deconjugation rate and metabolic conversion, are outcomes of multiple factors. These factors include solvent accessibility, local pKa shifts, polarity, and the tendency to form complexes with serum proteins. Needless to say, linker design and engineering can play a critical role, in addition to conjugation site and chemistry. There has been significant advancement in linker design, which continues to evolve and advance ADC therapeutics.^[^ ^14^, ^20^ ^]^ This progress highlights the need for a comprehensive approach to ADC development, taking into account the interplay of various factors to optimize therapeutic efficacy and stability. Herein, we describe the effect of four different conjugation modes/chemistries: lysine, hinge‐cysteine, bTG‐mediated site‐specific, and cysteine‐engineered site‐specific conjugation on ADC biotransformation (payload metabolism and payload‐linker deconjugation) and ultimately the ADC stability and pharmacokinetics.

## 4 Experimental Section

4.1

4.1.1

##### In Vivo Efficacy and Pharmacokinetics of ADCs

The in vivo efficacy of the lysine, hinge‐cysteine, and site‐specific ADCs was evaluated in N87 gastric xenograft tumor model. Each C.B–17 SCID mice (7–8 weeks old) was implanted with 5 million tumor cells in 50% Matrigel on both flanks. One week after implantation, the animals were randomized into different groups and dosed via retro‐orbital injection with specific ADCs (0.25, 0.5, 1, and 3.5 mg/kg, single dose). Tumor volume and body weight changes were monitored and recorded periodically. Serial blood samples were collected at 5 min, 4, 24, 72, 168, and 336 h postinjection for pharmacokinetic analysis. For cysteine‐engineered site‐specific ADCs, the ADCs were dosed at 2 mg/kg in nontumor‐bearing SCID mice, and serial blood samples were collected only for PK analysis.

##### Quantification of Total Ab, Total ADC, Active ADC in PK Samples

All three LBA were performed using a sandwich immunoassay format on Bioaffy 200 microfluidic CDs containing streptavidin‐coated columns loaded onto a Gyrolab xP platform (Gyros Protein Technologies, Uppsala, Sweden). The capture and detect reagents used in the LBA assays were generated at Bristol‐Myers Squibb. The capture reagent for all the three analytes was a biotinylated mouse anti‐idiotype (anti‐ID) monoclonal antibody. For the total Ab assay, the detection reagent was an Alexa‐647 conjugated mouse anti‐human IgG Fc mAb (Bristol‐Myers Squibb). For the total ADC assay, the detection reagent was an Alexa‐647 conjugated mouse antipayload antibody (LBA‐R1) that binds to both active and inactive forms of the payload. For the active ADC assay, the detection reagent was an Alexa‐647 conjugated mouse antipayload antibody (LBA–R2) that binds only to the active form of the payload and does not bind to inactive form. A four‐parameter logistic regression curve with 1/y2 weighting was used to calculate the concentrations of T‐Ab, T‐ADC, and A‐ADC in the samples.

##### In Vitro Serum Stability Assessment

Anti‐MSLN (Cysteine Engineered) ADCs were added to SCID serum at a concentration of 50 μg/ml and incubated at 37 °C. Serum aliquots were collected at 0, 24, 48, and 96 h postincubation. The samples were then analyzed by affinity capture LC‐MS method recently reported (Kotapati 2020),^[^ ^18^ ^]^ and the MS data were analyzed after deconvolution using the Intact Mass software (v3.6, Protein Metrics, Inc.). The peak intensities of the parent ADC and the catabolite species (hydrolysis, deacetylation, etc.) were used to determine the % succinimide hydrolysis, % deacetylation, and % deconjugation.

##### Pharmacokinetic Data Analysis

The total Ab, total ADC, and active ADC concentrations measured from serum were used to generate PK profiles of these analytes. Noncompartmental analyses were performed with Phoenix WinNonlin version 6.4 (Certara, St. Louis, Missouri, USA). The area under the serum concentration‐time curve (AUC) was determined by the log‐linear trapezoidal rule. Finally, the following PK parameters were generated: highest concentration observed (Cmax), area under the curve from 0 to the last time point (AUC0‐336 h), half‐life (T1/2), and clearance (Cl_F).

## Conflict of Interest

The authors declare no conflict of interest.

## Supporting information

Supplementary Material

## References

[1] Z. Fu , S. Li , S. Han , C. Shi , Y. Zhang , Signal Transduct. Target. Ther. 2022, 7, 93. 35318309 10.1038/s41392-022-00947-7 PMC8941077

[2] P. Khongorzul , C. J. Ling , F. U. Khan , A. U. Ihsan , J. Zhang , Mol. Cancer Res. 2020, 18, 3. 31659006 10.1158/1541-7786.MCR-19-0582

[3] K. Tsuchikama , Z. An , Protein. Cell. 2018, 9, 33. 27743348 10.1007/s13238-016-0323-0 PMC5777969

[4] J. R. McCombs , S. C. Owen , AAPS J. 2015, 17, 339. 25604608 10.1208/s12248-014-9710-8 PMC4365093

[5] a) S. Panowski , S. Bhakta , H. Raab , P. Polakis , J. R. Junutula , MAbs 2014, 6, 34; 24423619 10.4161/mabs.27022 PMC3929453

[6] a) J. R. Junutula , H. Raab , S. Clark , S. Bhakta , D. D. Leipold , S. Weir , Y. Chen , M. Simpson , S. P. Tsai , M. S. Dennis , Y. Lu , Y. G. Meng , C. Ng , J. Yang , C. C. Lee , E. Duenas , J. Gorrell , V. Katta , A. Kim , K. McDorman , K. Flagella , R. Venook , S. Ross , S. D. Spencer , W. Lee Wong , H. B. Lowman , R. Vandlen , M. X. Sliwkowski , R. H. Scheller , P. Polakis , W. Mallet , Nat. Biotechnol. 2008, 26, 925; 18641636 10.1038/nbt.1480

[7] a) S. Jeger , K. Zimmermann , A. Blanc , J. Grunberg , M. Honer , P. Hunziker , H. Struthers , R. Schibli , Angew. Chem., Int. Ed. Engl. 2010, 49, 9995; 21110357 10.1002/anie.201004243

[8] a) J. R. Junutula , K. M. Flagella , R. A. Graham , K. L. Parsons , E. Ha , H. Raab , S. Bhakta , T. Nguyen , D. L. Dugger , G. Li , E. Mai , G. D. Lewis Phillips , H. Hiraragi , R. N. Fuji , J. Tibbitts , R. Vandlen , S. D. Spencer , R. H. Scheller , P. Polakis , M. X. Sliwkowski , Clin. Cancer Res. 2010, 16, 4769; 20805300 10.1158/1078-0432.CCR-10-0987

[9] a) A. Kaempffe , S. Dickgiesser , N. Rasche , A. Paoletti , E. Bertotti , I. De Salve , F. R. Sirtori , R. Kellner , D. Konning , S. Hecht , J. Anderl , H. Kolmar , C. Schroter , J. Pharm. Sci. 2021, 110, 3776; 34363839 10.1016/j.xphs.2021.08.002

[10] a) S. C. Alley , D. R. Benjamin , S. C. Jeffrey , N. M. Okeley , D. L. Meyer , R. J. Sanderson , P. D. Senter , Bioconjug. Chem. 2008, 19, 759; 18314937 10.1021/bc7004329

[11] C. F. McDonagh , E. Turcott , L. Westendorf , J. B. Webster , S. C. Alley , K. Kim , J. Andreyka , I. Stone , K. J. Hamblett , J. A. Francisco , P. Carter , Protein Eng., Des. Sel. 2006, 19, 299. 16644914 10.1093/protein/gzl013

[12] L. N. Tumey , C. A. Leverett , B. Vetelino , F. Li , B. Rago , X. Han , F. Loganzo , S. Musto , G. Bai , S. C. Sukuru , E. I. Graziani , S. Puthenveetil , J. Casavant , A. Ratnayake , K. Marquette , S. Hudson , V. R. Doppalapudi , J. Stock , L. Tchistiakova , A. J. Bessire , T. Clark , J. Lucas , C. Hosselet , C. J. O’Donnell , C. Subramanyam , ACS Med. Chem. Lett. 2016, 7, 977. 27882194 10.1021/acsmedchemlett.6b00195 PMC5108037

[13] M. O’Hara , C. Stashwick , A. R. Haas , J. L. Tanyi , Immunotherapy 2016, 8, 449. 26973126 10.2217/imt.16.4 PMC5619020

[14] Y. Anami , C. M. Yamazaki , W. Xiong , X. Gui , N. Zhang , Z. An , K. Tsuchikama , Nat Commun. 2018, 9, 2512. 29955061 10.1038/s41467-018-04982-3 PMC6023893

[15] J. Clarke , S.‐C. Chu , L.L. Siu J.‐P. Machiels , B. Markman , K. Heinhuis M. Millward , M. Lolkema , S. P. Patel P. de Souza , G. Curigliano , A. Santoro , M. Brown , R. Fleming , H. Vezina , C. He , S. Rottey , Mol. Cancer Ther. 2019, 18, B057.

[16] a) B. Q. Shen , K. Xu , L. Liu , H. Raab , S. Bhakta , M. Kenrick , K. L. Parsons‐Reponte , J. Tien , S. F. Yu , E. Mai , D. Li , J. Tibbitts , J. Baudys , O. M. Saad , S. J. Scales , P. J. McDonald , P. E. Hass , C. Eigenbrot , T. Nguyen , W. A. Solis , R. N. Fuji , K. M. Flagella , D. Patel , S. D. Spencer , L. A. Khawli , A. Ebens , W. L. Wong , R. Vandlen , S. Kaur , M. X. Sliwkowski , R. H. Scheller , P. Polakis , J. R. Junutula , Nat. Biotechnol. 2012, 30, 184; 22267010 10.1038/nbt.2108

[17] S. Yamazoe , S. Kotapati , J. M. Hogan , S. M. West , X. A. Deng , S. J. Diong , J. Arbanas , T. A. Nguyen , A. Jashnani , D. Gupta , A. Rajpal , G. Dollinger , P. Strop , Bioconjug. Chem. 2022, 33, 576. 35344340 10.1021/acs.bioconjchem.1c00572 PMC9026278

[18] S. Kotapati , D. Passmore , S. Yamazoe , R. K. K. Sanku , Q. Cong , Y. B. Poudel , N. S. Chowdari , S. Gangwar , C. Rao , V. S. Rangan , P. M. Cardarelli , S. Deshpande , P. Strop , G. Dollinger , A. Rajpal , Anal. Chem. 2020, 92, 2065. 31860282 10.1021/acs.analchem.9b04572

[19] L. N. Tumey , F. Li , B. Rago , X. Han , F. Loganzo , S. Musto , E. I. Graziani , S. Puthenveetil , J. Casavant , K. Marquette , T. Clark , J. Bikker , E. M. Bennett , F. Barletta , N. Piche‐Nicholas , A. Tam , C. J. O’Donnell , H. P. Gerber , L. Tchistiakova , AAPS J. 2017, 19, 1123. 28439809 10.1208/s12248-017-0083-7

[20] a) T. Watanabe , N. Arashida , T. Fujii , N. Shikida , K. Ito , K. Shimbo , T. Seki , Y. Iwai , R. Hirama , N. Hatada , A. Nakayama , T. Okuzumi , Y. Matsuda , J. Med. Chem. 2024, 67, 18124; 39410752 10.1021/acs.jmedchem.4c01251 PMC11513888

