# Supplementary material for: The Impact of Conjugation Mode and Site on Tubulysin Antibody‐Drug‐Conjugate Efficacy and Stability
Source: ChemistryOpen. 2025 May 28;14(8):e202400522. doi: 10.1002/open.202400522 (PMC12368874; doi:10.1002/open.202400522)
Supplement: Supplementary file 1 — Supplementary Material [file OPEN-14-e202400522-s001.pdf]

SUPPORTING INFORMATION

Synthesis

Synthesis of tubulysin and PBD for bTGase and cysteine conjugation is described in the patents (US10279049B2, WO 2016115191).

Preparation of the three ADCs

Anti-MSLN ADC made with lysine modification (random ADC)

Fresh solution of 10 mM 2-iminothiolane (2-IT) in water was prepared and a 20-fold molar excess was added to the anti-MSLN antibody (5mg/ml in 1x PBS, pH 8.0). The reaction was carried out for 1.5 h at room temperature by gentle stirring. The thiolated antibody was buffer exchanged into 50 mM HEPES, 5 mM glycine, 2 mM DTPA, pH 6.05 by tangential glow filtration (TFF). The number of thiols were measured using a dithiodipyridine (DTDP) assay. The payload linker (**1**, **2** or **4**) in DMSO was added to the thiolated antibody in an equimolar amount per thiol of antibody. The reaction mixture was gently mixed at room temperature for 1.5 h. To block any unreacted thiols, N-Ethyl Maleimide (NEM) was added at a 10-fold molar excess per thiol of antibody. The ADC was purified and buffer exchanged from the unconjugated drug and other added components by TFF into 20 mM Histidine, 10% Sucrose, pH 6.0 buffer.

Anti-MLSN ADC made with hinge cysteine modification

Anti-MSLN mAb (5 mg/mL) was partially reduced with 2.2 fold molar excess of TCEP at 37°C.The number of thiols/Ab was determined using a DTDP assay. The payload linker (**1**) was added to the above antibody at a 1.25 molar excess to thiols per Ab. After the addition of the drug, the reaction mixture was incubated at 4°C for 2 h. This was followed by incubation of the ADC with N-acetyl-cysteine at 10-fold molar excess of payload-linker to block any unreacted drug. This reaction is further incubated for1 h at 4 0C. The ADC was buffer exchanged using TFF into 20 mM Histidine, 10% Sucrose, pH 6.0 buffer.

Anti-MSLN ADC made with bTGase

Anti-MSLN mAb with N297Q/N297A mutation (5mg/mL) in Tris, pH 8.0 buffer was incubated with recombinant bacterial transglutaminase (1/5 mol equivalent) and 15 mole equivalent of payload Linker (**3** or **5**). The reaction was carried out at 37°C overnight. The ADC was purified on a Protein A column using standard protocol. The purified ADC was formulated in 20 mM Histidine, 10% Sucrose, pH 6.0 buffer.

Anti-MSLN ADCs made with introducing unpaired cysteine

Anti-MSLN cysteine engineered mutant antibodies (~ 5mg/mL) were subjected to mild reduction in 50 mM Tris-HCl, pH7.5, 2 mM EDTA at 37 ^{0}C by the addition of 10-fold molar (per antibody) excess of TCEP for 2 hours. The inter-chain disulfide reduction was monitored by RP-UPLC. The excess TCEP was removed by PD-mini G25 column. Buffer was exchanged to 1x PBS pH7.4. DTDP assay was performed to determine the number of thiol/mAb. The re-formation of the interchain disulfide was conducted with incubation with 15-fold excess of dhAA. The mixture was incubated at 25 ^{0}C for 3 hrs. The re-oxidation reaction was monitored by DTDP assay or RP-HPLC until 2 thiol/mAb or intact mAb and disappearance of LC and HC. 3 molar excess of payload-linker (**1**) was incubated with the activated antibody for 1 hr at 25 ^{0}C. Free-drug removal and formulation was performed by TFF. The conjugation reaction mixture is buffer exchanged against 10x volume of 20 mM Histidine, 10% Sucrose pH6.0. The filter used is Vivaflow 50R from Sartorius with a 30,000 MWCO hydrosart membrane.

Cellular binding of the ADCs and naked antibodies

Cells were detached using Corning® 100 mL Cellstripper™ and resuspended in 50 μL of FACS buffer (1x PBS with 2% heat-inactivated FBS and 0.1% sodium azide). A 2× stock solution of each antibody/ADC was prepared and serially diluted in a 1:4 ratio before being mixed with the cells. After incubation for 1 hour at 4°C, the cells were washed with buffer and incubated with the secondary antibody (Jackson ImmunoResearch Labs Alexa Fluor 647 AffiniPure Fab Fragment Goat Anti-Human IgG, Fcγ fragment specific) for 30 minutes at 4°C. The cells were then washed with buffer and analyzed using a FACS machine (Beckman CytoFLEX).

Cytotoxicity assay of the ADCs

Cells were seeded in 100 μL of RPMI-1640 medium with 10% FBS into 96-well flat-bottomed plates at the following densities: 1500 cells/well for H226, 2000 cells/well for N87, 3000 cells/well for OVCAR3 (in RPMI-1640 supplemented with 20% FBS and insulin), and 150 cells/well for CHO K1. The cells were incubated at 37 °C for 4 hours to allow for

adherence. A 6× stock solution of each ADC was prepared and serially diluted in a 1:4 ratio before being added to the cells. After drug treatment, 25 μL of additional growth media was added. The treated cells were then cultured at 37 °C for 7 days. Cell viability was measured using the Cell Titer-Glo (CTG) Luminescent Viability Assay from Promega. To each well, 100 μL of reconstituted CTG reagent was added, followed by gentle shaking for 10 minutes. Luminescence was measured using the Cytation 5 (Biotek Instruments, Winooski, VT). The percentage of cell viability was calculated using the formula: (average luminescence of treated samples / average luminescence of untreated control samples) × 100. EC50 values were calculated using logistic nonlinear regression analysis with Prism v7.02 software (GraphPad, San Diego, CA).

Table S1: Nonclinical Pharmacokinetics: Absorption, Distribution, and Metabolism Data of Lysine ADC targeting Meso

Species	Route/Strain	Animals per group (M/F)	Mean Parameters				
			AUC(NF) (ug*h/mL)	T1/2 (h)	MRT (h)	CLT (mL/h/kg)	Vss (L/kg)
Mouse/SCID	IV/10	3 per timepoint (M)	8700	100	99	1.2	0.11
Mouse/SCID	IV/7.4	3 per timepoint (M)	5250	95	99	1.4	0.14
Rat	IV/5	10 (M and F)	5250	130	131	0.94	0.12
Rat	IV/7.6	10 (M and F)	7500	120	116	1.01	0.12
Monkey	IV/2.5	5 (M and F)	4950	79	101	0.51	0.052
Monkey	IV/5	5 (M and F)	11500	140	146	0.43	0.063

Figure S1. Cellular binding characteristics of naked antibody and ADCs

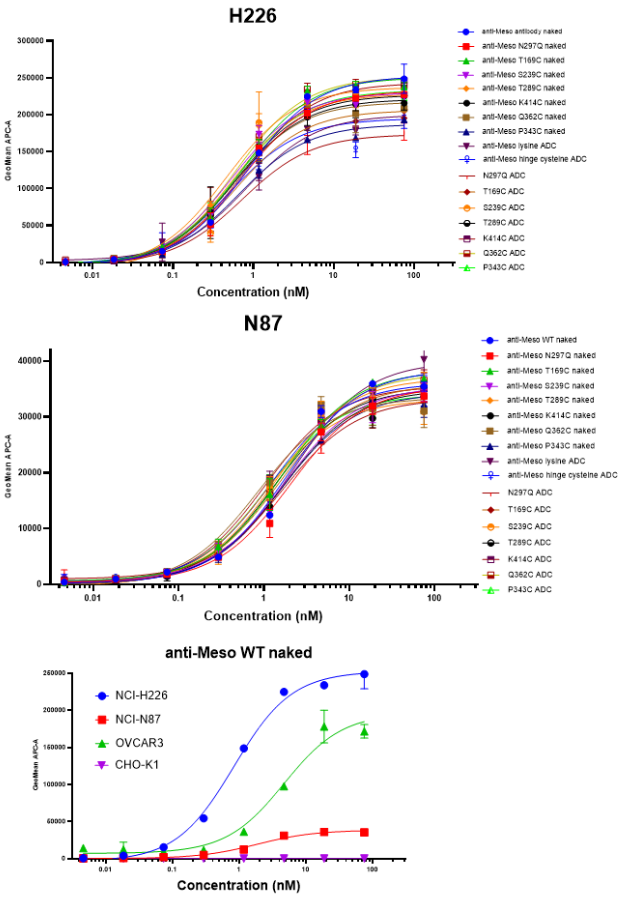

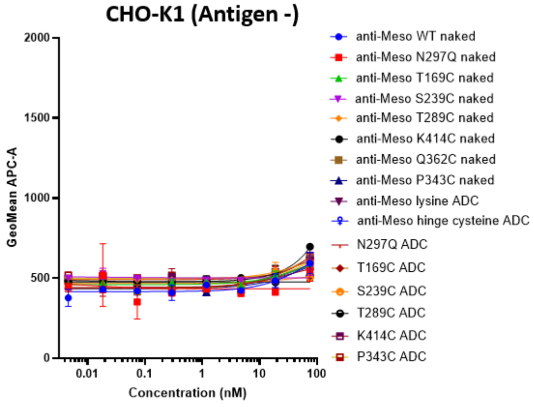

	NCI-H226 EC50 (nM)	NCI-N87 EC50 (nM)
WT naked	0.8324	1.832
N297Q naked	0.7092	2.045
T169C naked	0.6989	1.617
S239C naked	0.5128	1.705
T289C naked	0.4555	1.366
K414C naked	0.5766	1.671
Q362C naked	0.5489	0.8507
P343C naked	0.6487	1.625
Anti-Meso lysine ADC	0.8285	2.121
Anti-Meso hinge cysteine ADC	0.5182	1.225
N297Q ADC	0.7219	1.684
T169C ADC	0.6117	1.505
S239C ADC	0.5262	1.013
T289C ADC	0.5898	1.301
K414C ADC	0.7481	1.034
Q362C ADC	0.5929	1.517
P343C ADC	0.6107	1.078

Figure S1. Cellular binding characteristics of naked antibody and ADCs

Figure S2. In vitro cell killing activity of anti-Meso (aMeso) ADCs bearing Tubulysin payload.

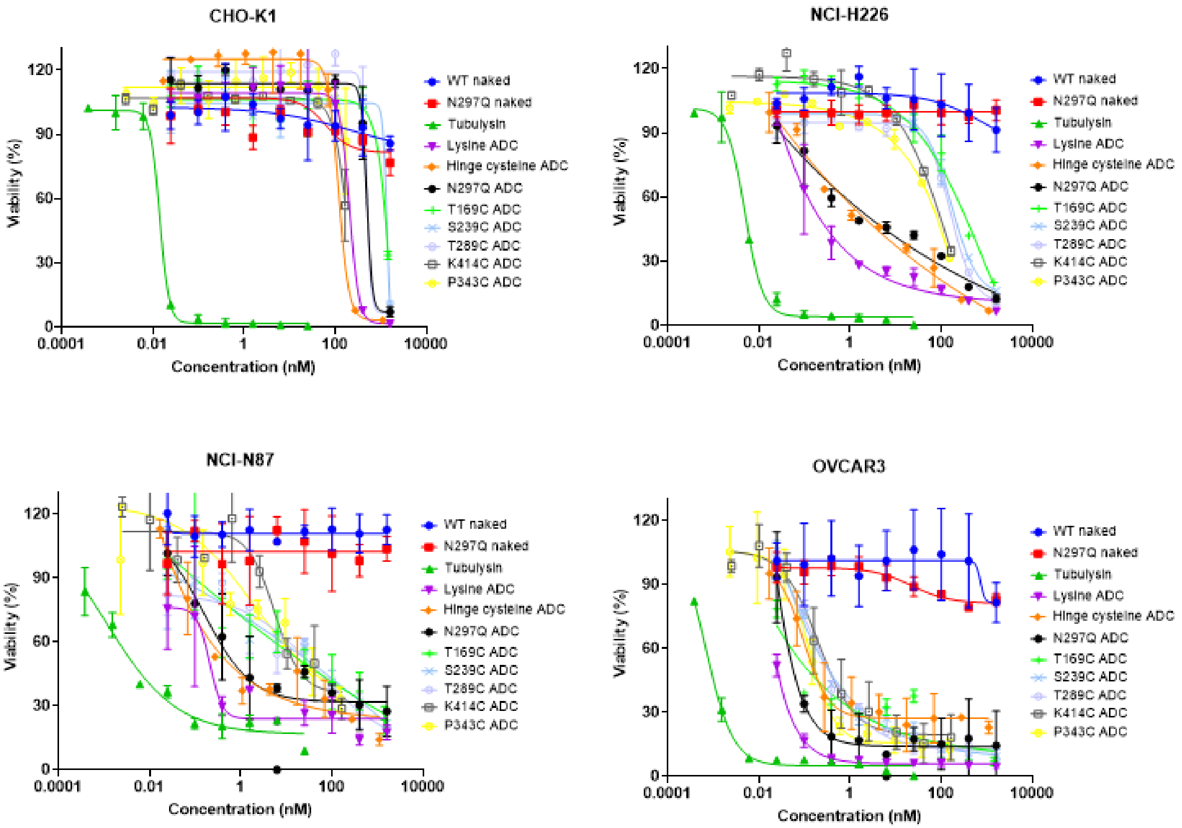

Figure S3. In vitro cell killing activity of anti-Meso (aMeso) ADCs bearing Tubulysin acetyl (**1**) or Tubulysin deacetyl (**2**)

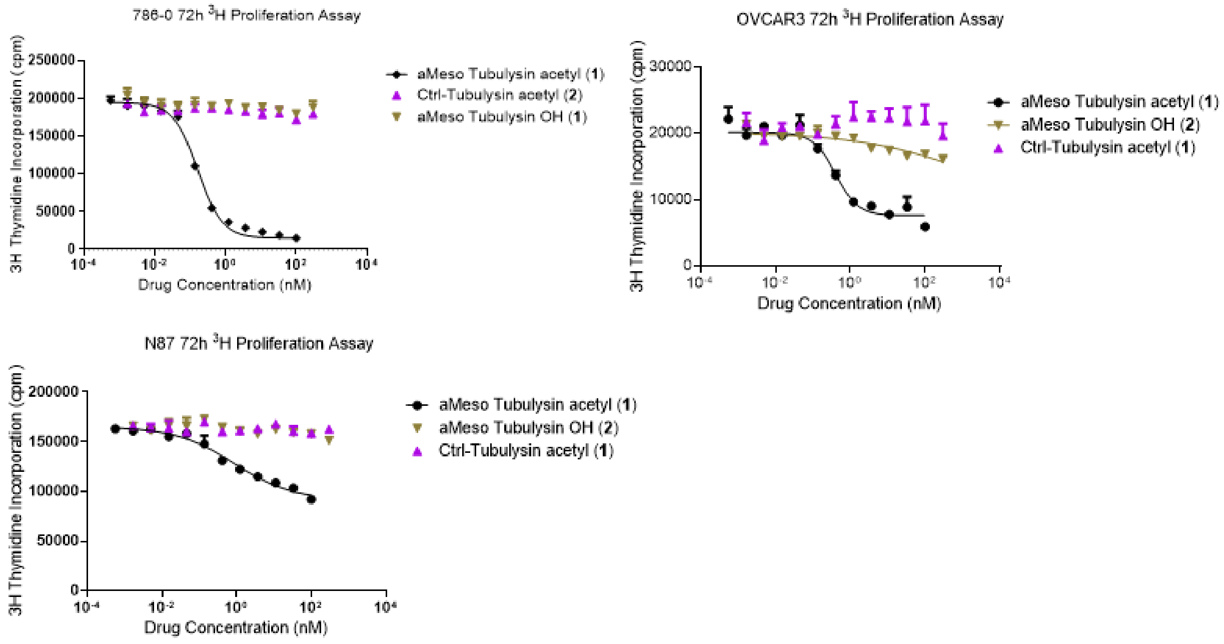

Figure S4. In vivo efficacy of anti-Meso (aMeso) ADCs bearing Tubulysin acetyl (**1**) or Tubulysin deacetyl (**2**). (a) Strucutures of linker payloads. (b) Efficacy of the ADCs in an N87 gastric xenograft model.

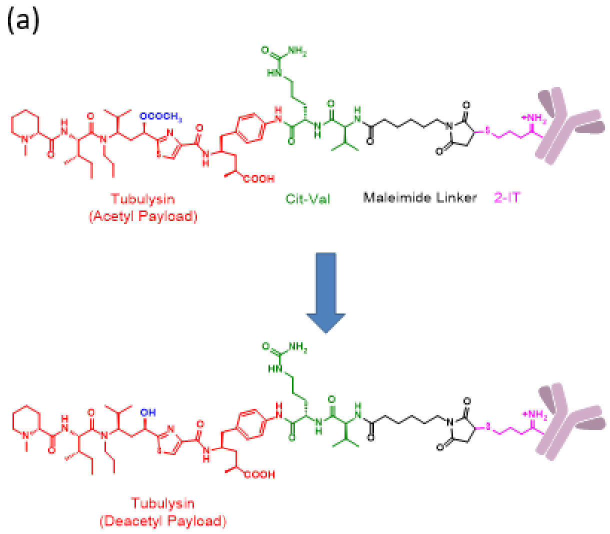

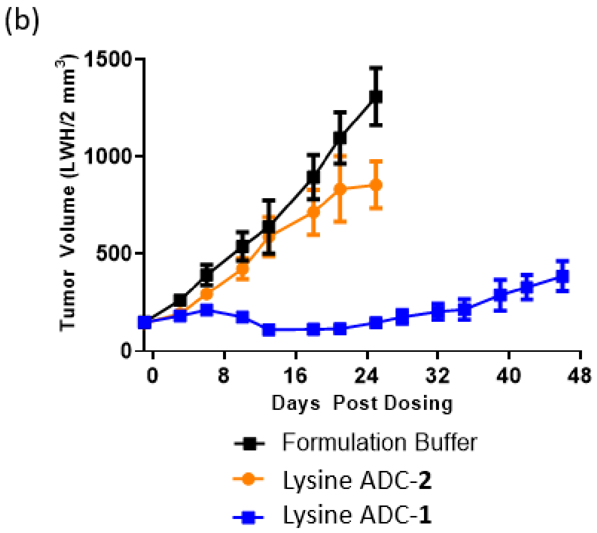

Figure S5. Body weight loss data after drug administration. No substantial difference between the three ADCs was observed.

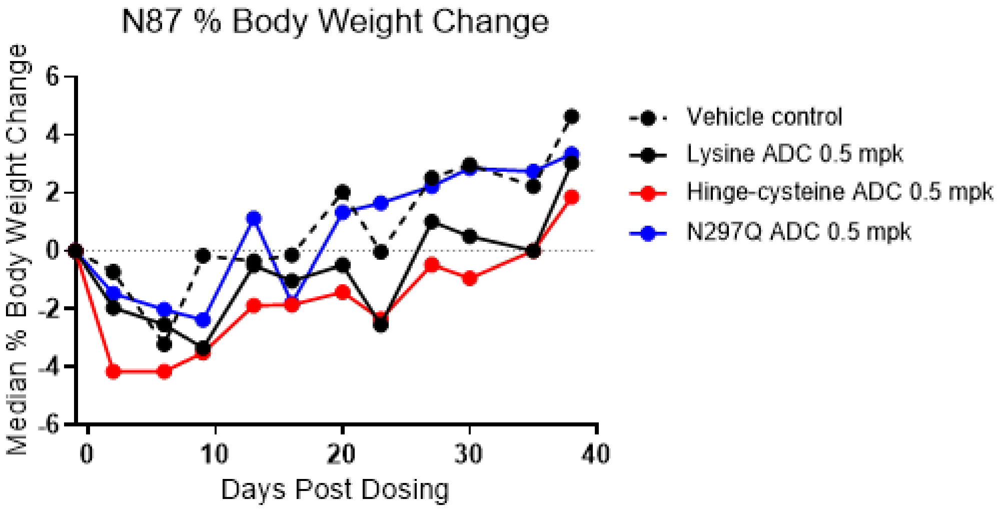

Figure S6. Binding characteristics of anti-payload antibodies

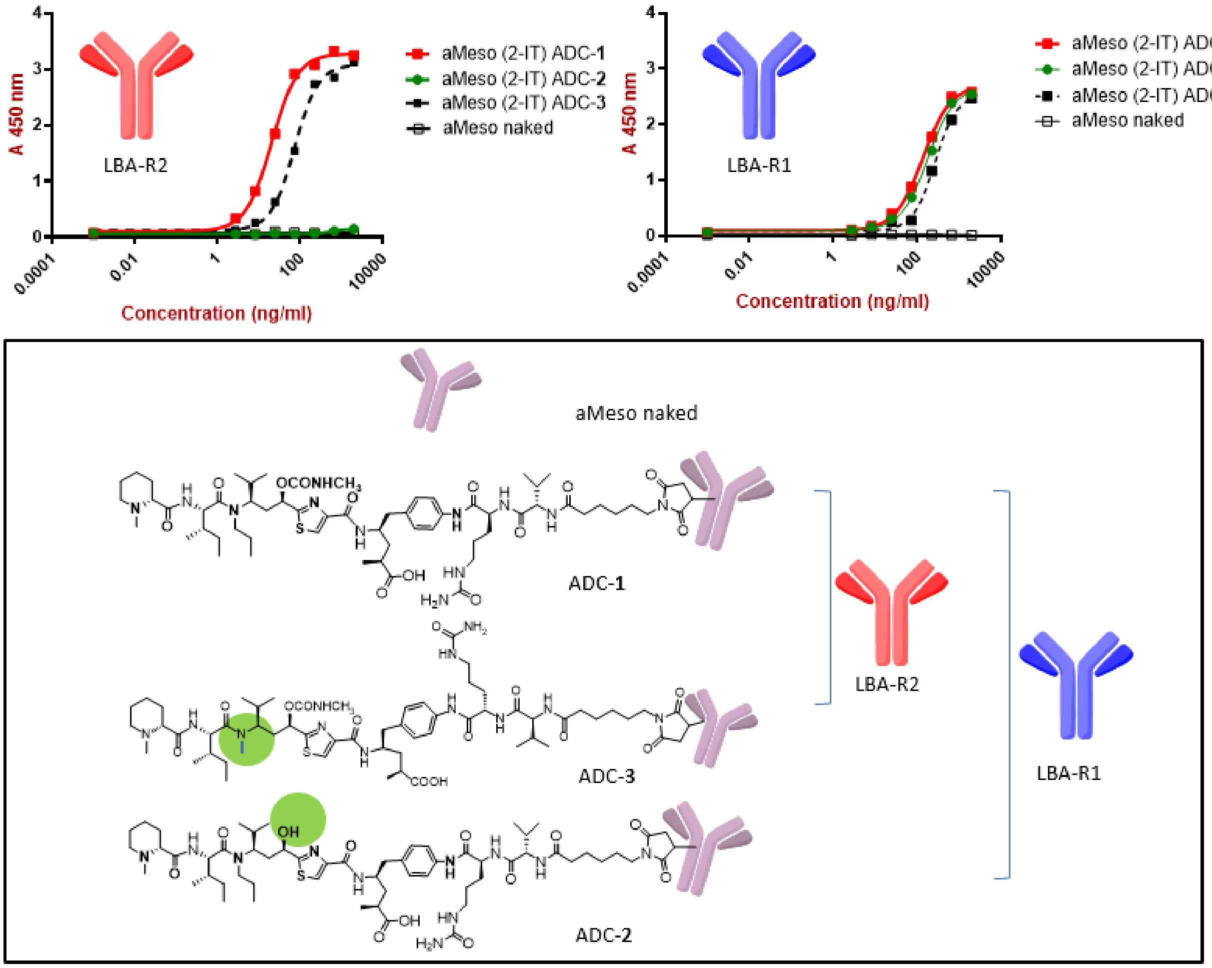

Figure S7. Mass spectrometric characterization of the Tubulysin ADCs to evaluate in vivo transformation

(a) Lysine ADC (Fab)

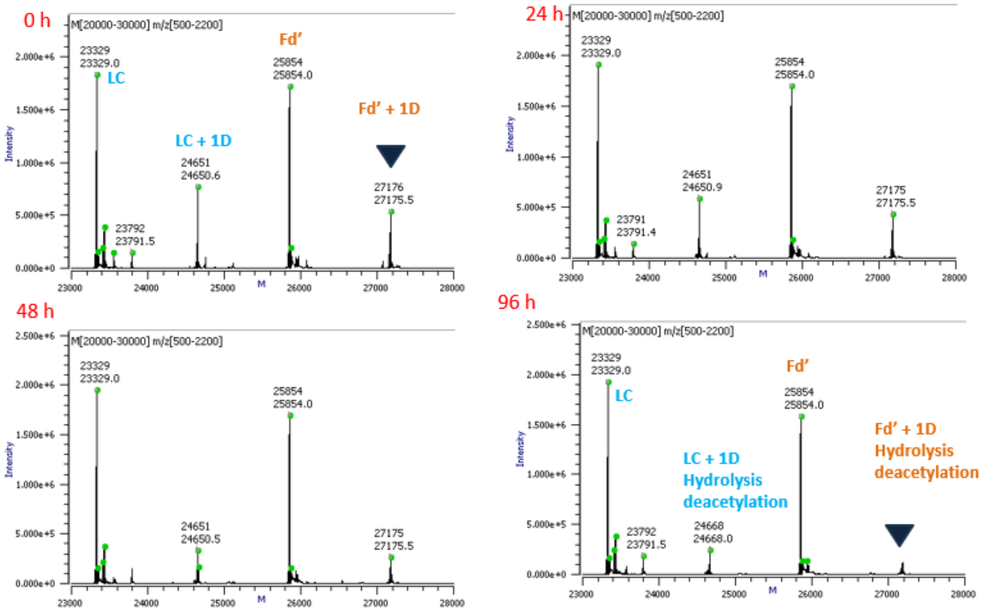

(b) Lysine ADC (Fc)

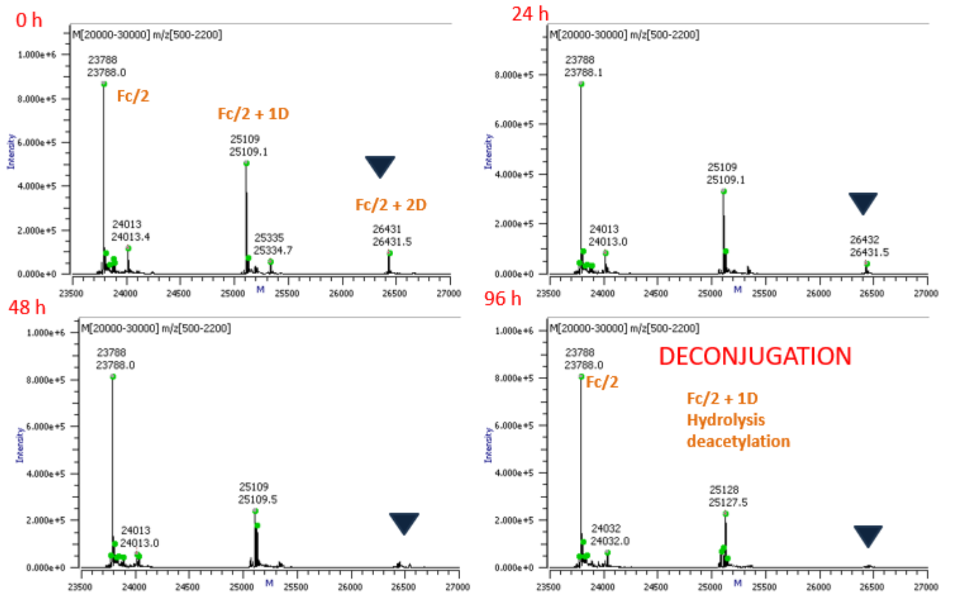

(c) Hinge cysteine ADC

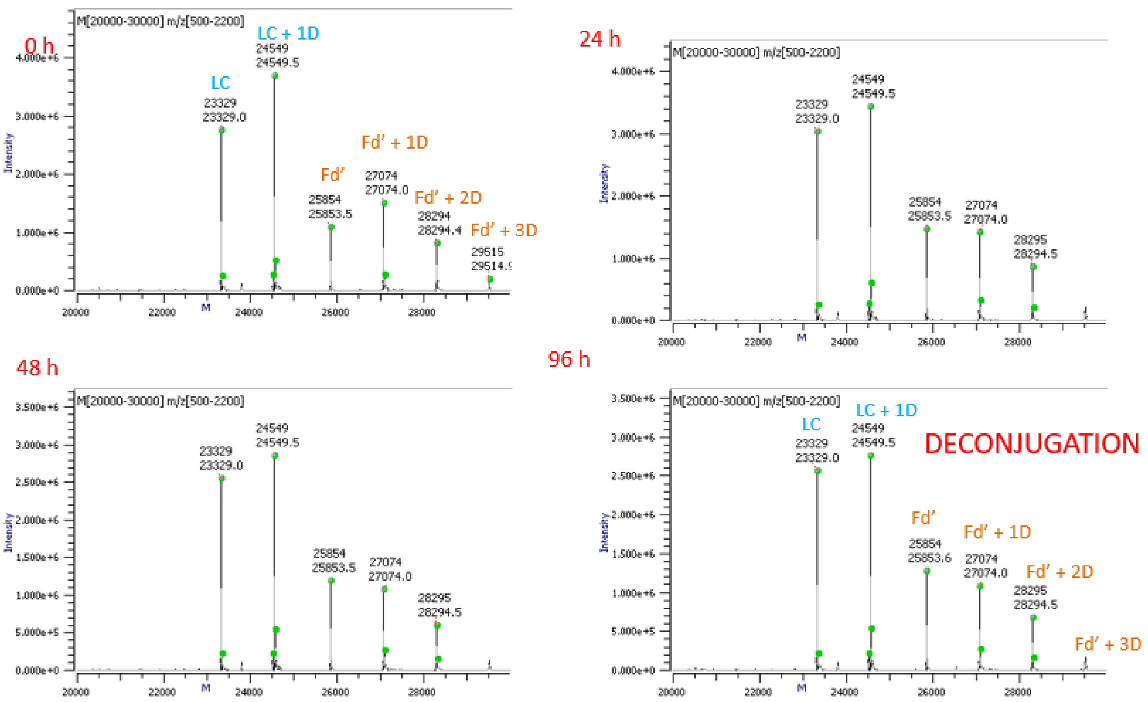

(d) N297Q ADC

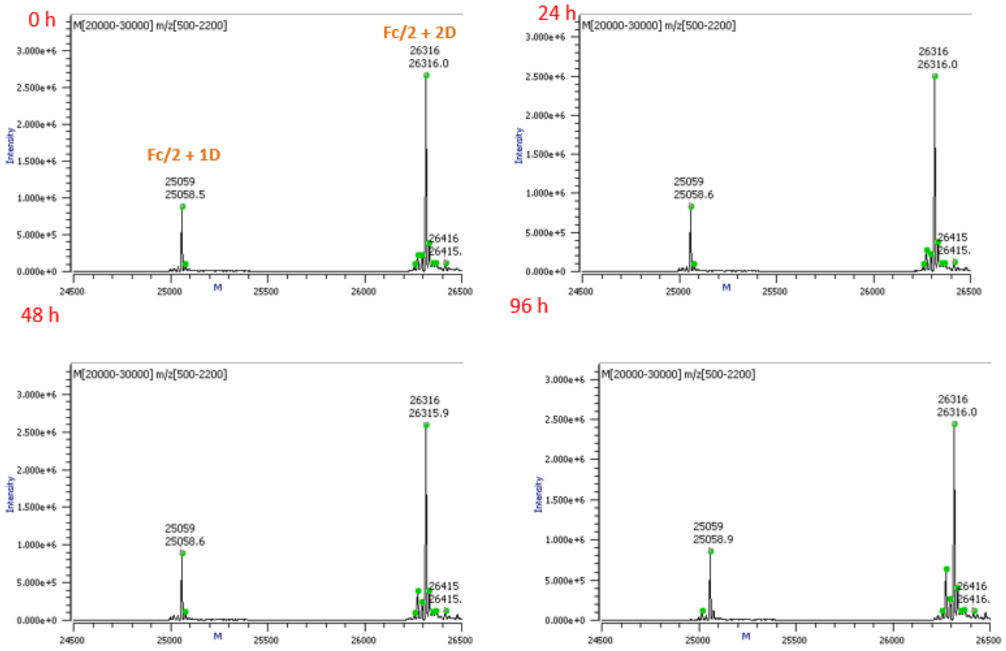

(e) T169C ADC

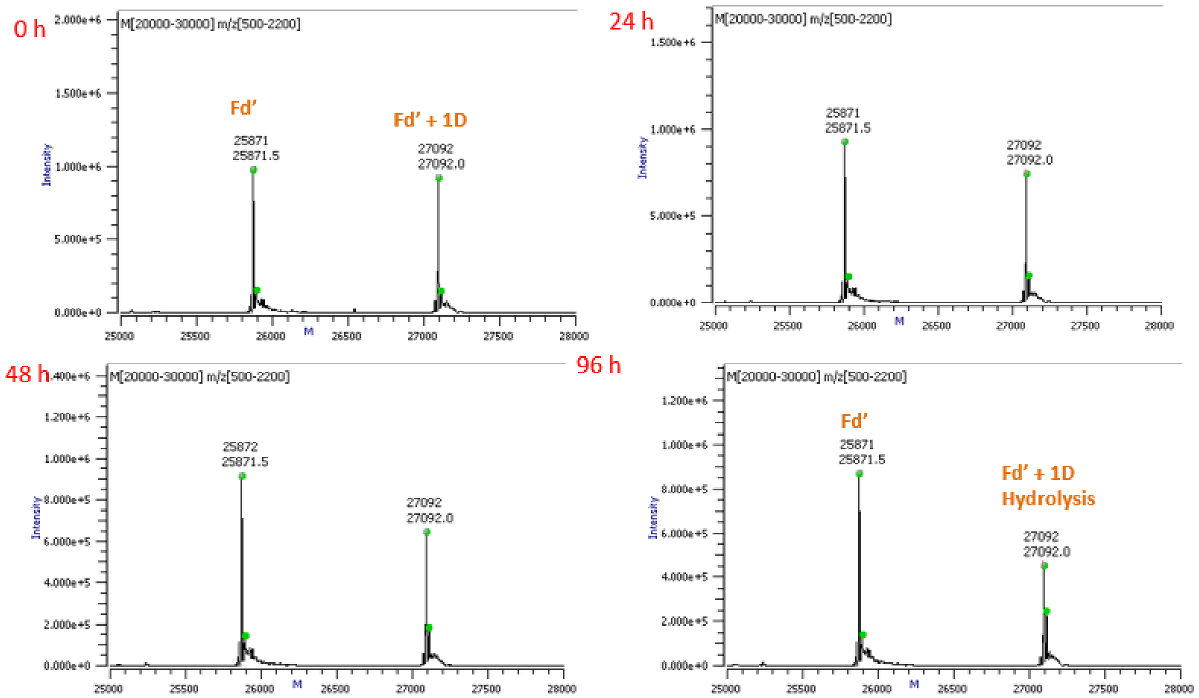

(f) S239C ADC

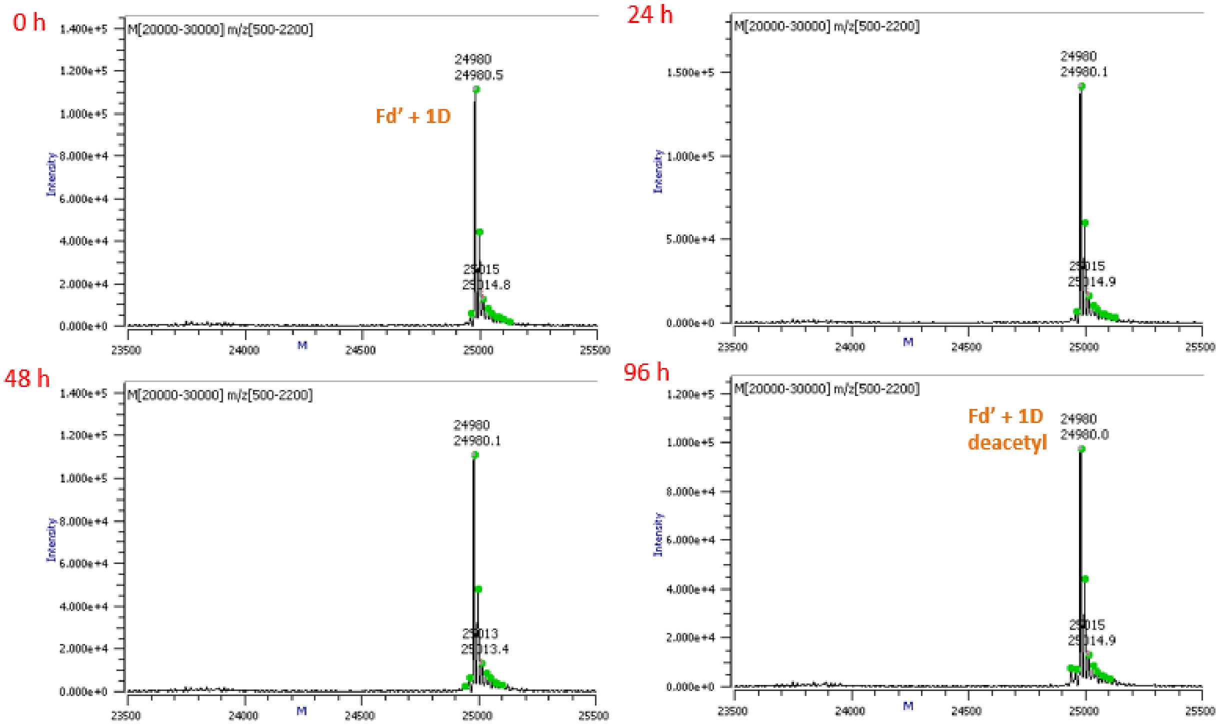

(g) T289C ADC

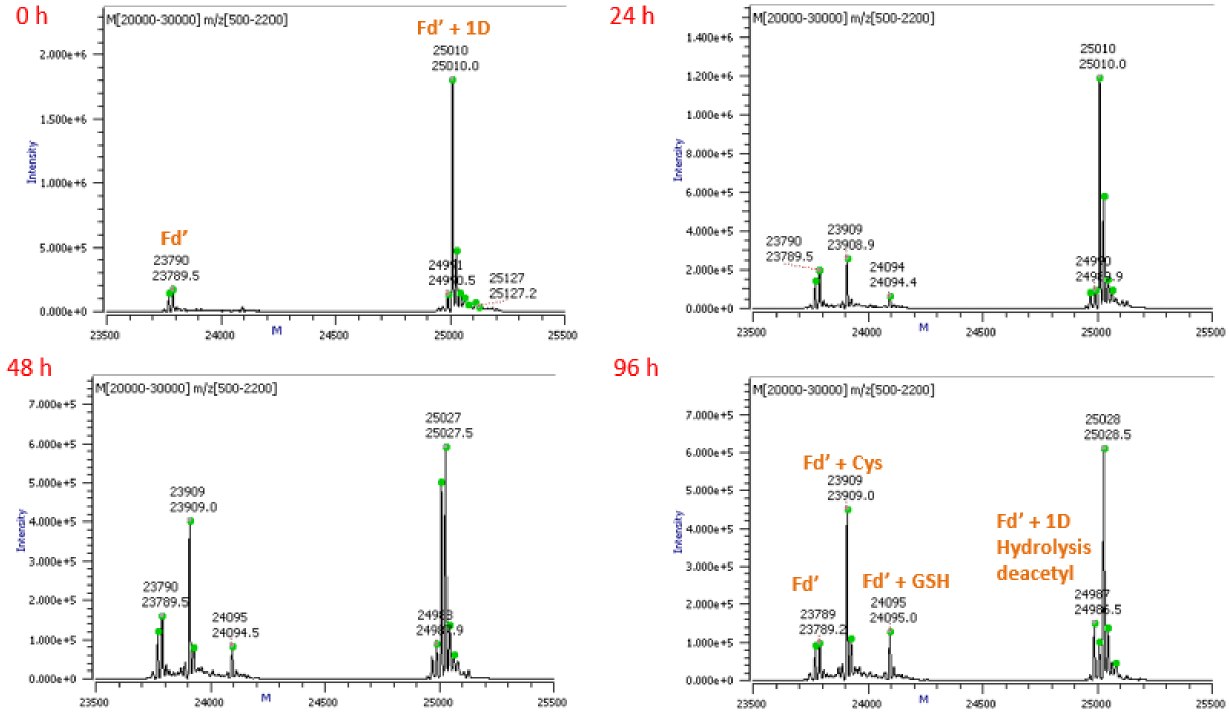

(h) P343C ADC

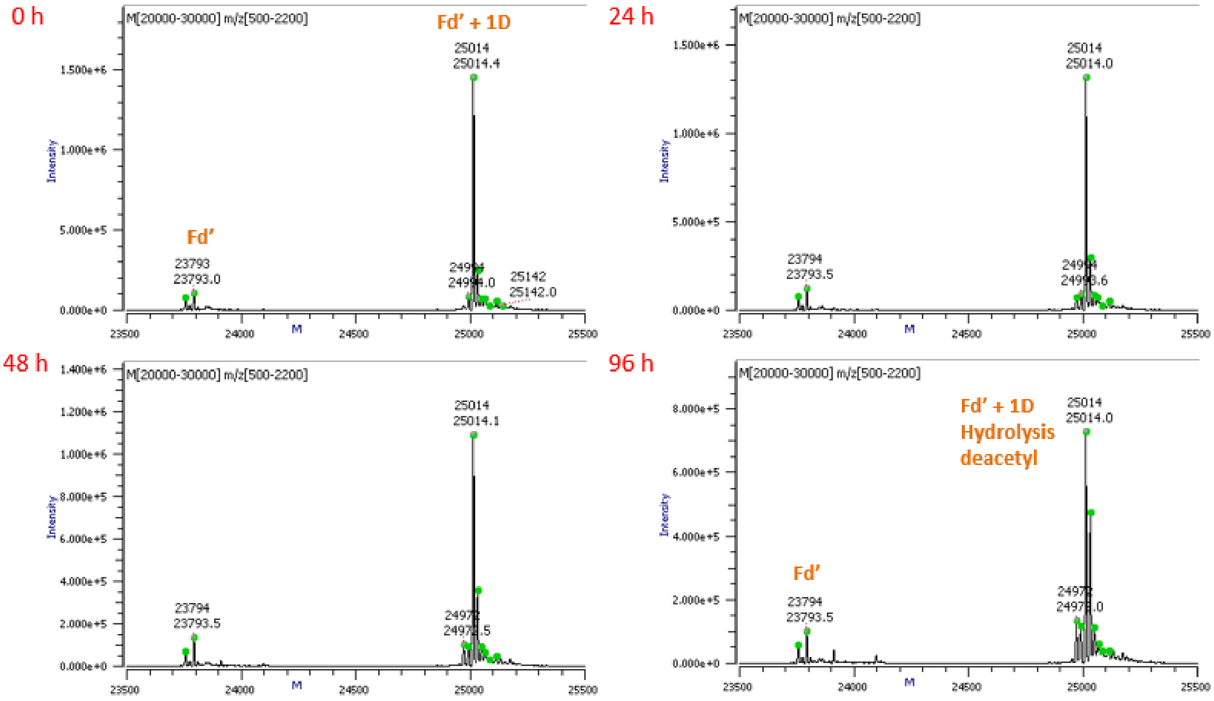

(i) Q362C ADC

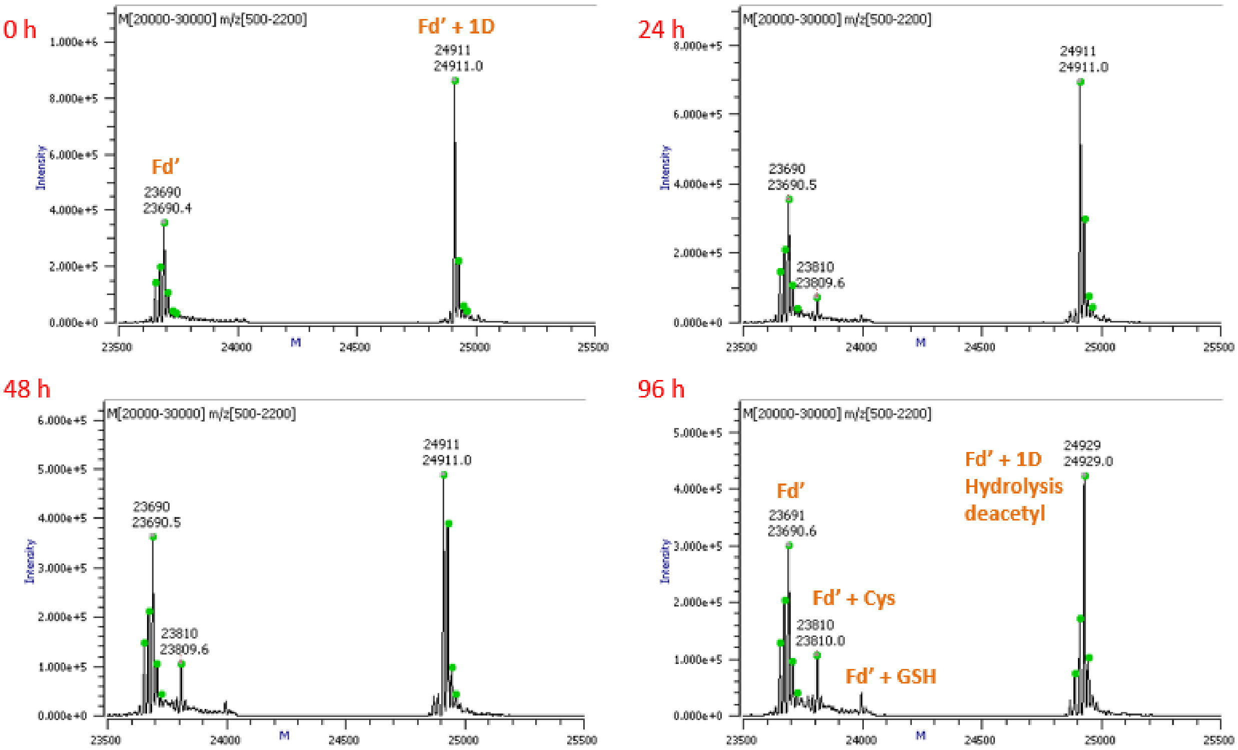

(j) K414C ADC

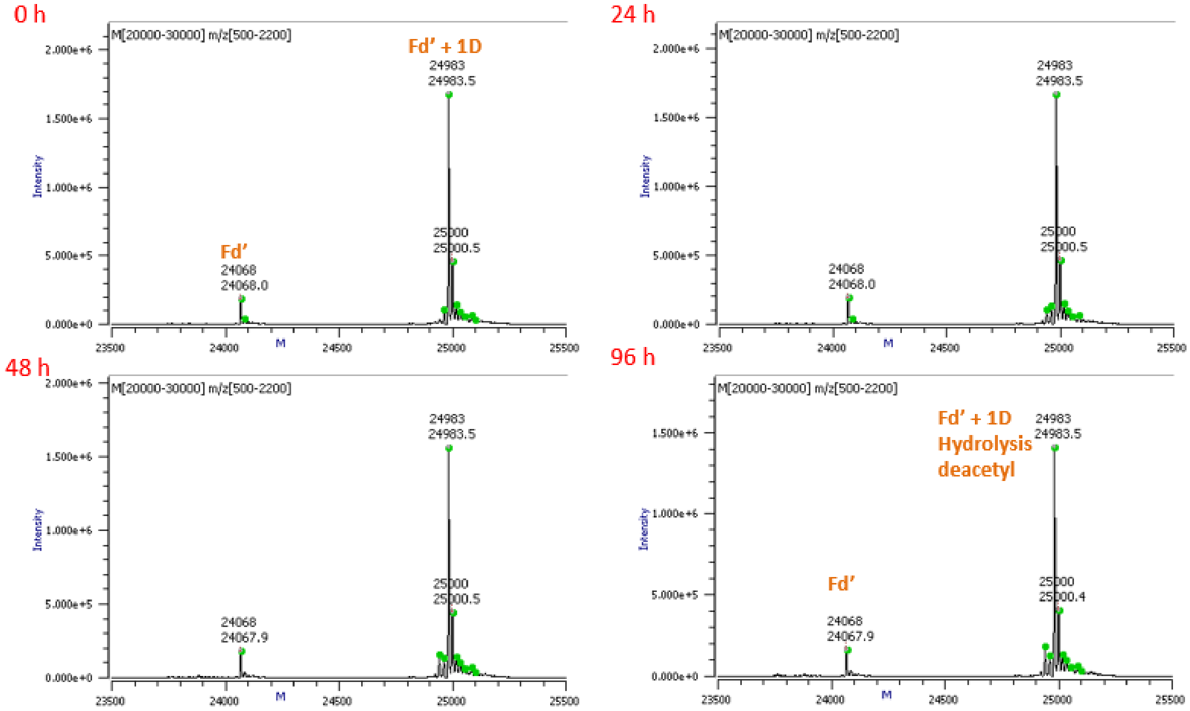

Figure S8. PK profiles of 6 cysteine-engineered site-specific ADCs with payload-linker conjugated at different positions T169C (A), S239C (b), T289C (c), P343C (d), Q362C (e), K414C (f)

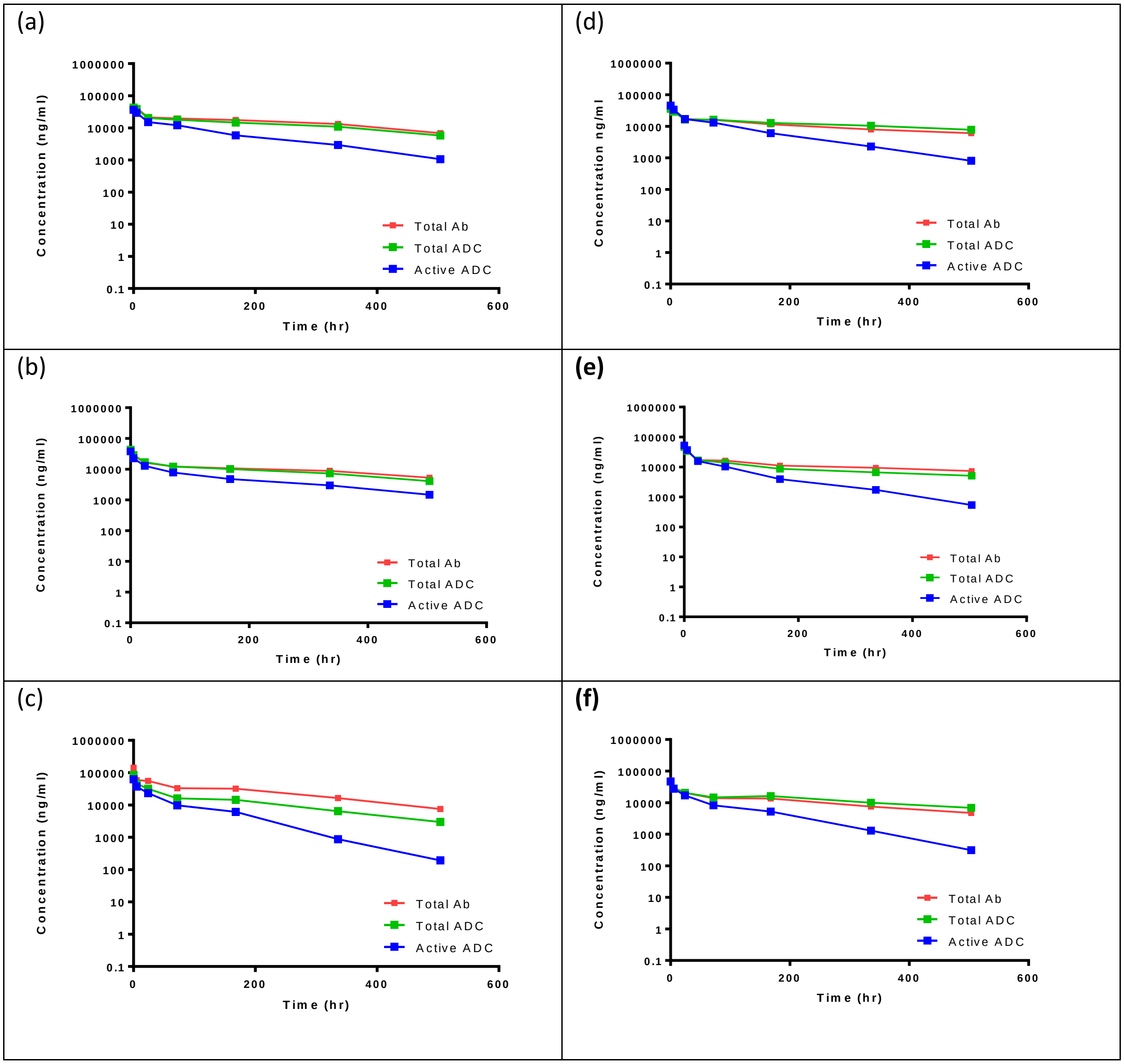

Figure S9. Structures of PBD dimer-based linker payloads. (a) PBD linker payload used for 2-IT conjugation. (b) PBD linker payload used for transglutaminase conjugation.

(a)

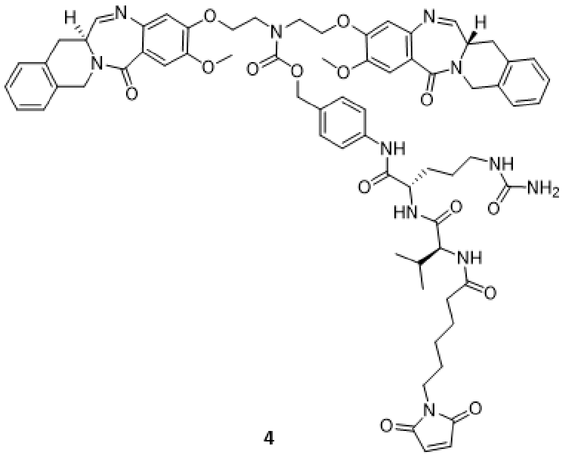

4

(b)

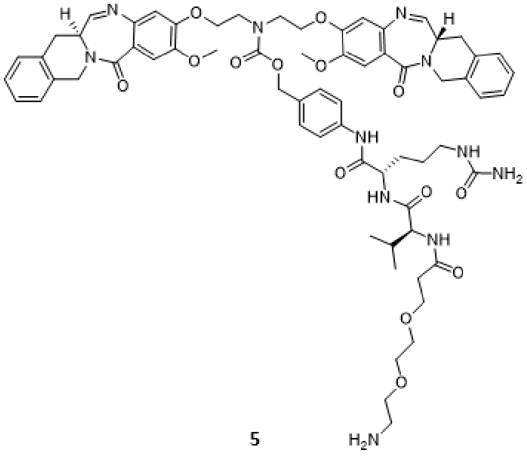

5
